# Fractal dimension of retinal vasculature as an image quality metric for automated fundus image analysis systems

**DOI:** 10.1038/s41598-022-16089-3

**Published:** 2022-07-13

**Authors:** Xingzheng Lyu, Purvish Jajal, Muhammad Zeeshan Tahir, Sanyuan Zhang

**Affiliations:** 1 College of Computer Science and Technology, Zhejiang University, 38 Zheda Road, Hangzhou, 310027 China; 2 Department of Electrical and Computer Engineering, University of Alberta, Edmonton, T6G 1H9 Canada

**Keywords:** Predictive markers, Disease prevention, Health services, Biomedical engineering

## Abstract

Automated fundus screening is becoming a significant programme of telemedicine in ophthalmology. Instant quality evaluation of uploaded retinal images could decrease unreliable diagnosis. In this work, we propose fractal dimension of retinal vasculature as an easy, effective and explainable indicator of retinal image quality. The pipeline of our approach is as follows: utilize image pre-processing technique to standardize input retinal images from possibly different sources to a uniform style; then, an improved deep learning empowered vessel segmentation model is employed to extract retinal vessels from the pre-processed images; finally, a box counting module is used to measure the fractal dimension of segmented vessel images. A small fractal threshold (could be a value between 1.45 and 1.50) indicates insufficient image quality. Our approach has been validated on 30,644 images from four public database.

## Introduction

Artificial intelligence powered automatic retinal image analysis (ARIA) systems such as detection of age-related macular degeneration and diabetic retinopathy (DR) could provide patients with ophthalmologist-like reports in seconds^1–6^. A well designed ARIA system usually requires high quality fundus images for guaranteed results and employs retinal image quality assessment (RIQA) to evaluate gradability of uploaded images^1,2,4,5^. In the largest public DR detection dataset EyePACS^7^, the number of ungradable images is estimated up to 25%^6^. Human and machine usually have limits of agreement over image quality when systems hit the ground^5^. Retinal images uploaded by nurses or camera technicians can often be rejected by the ARIA screening systems. Moreover, capturing images acceptable to such systems may take a long time, especially for unskilled human operators. In a word, RIQA is a subjective task for human but an objective one for computers.

The subjective RIQA metrics include image focus and clarity, artefacts, field definition and visibility of retinal anatomical structures (*i*.*e*. blood vessels, optic disk (OD) and macula)^8^. A comprehensive grading scale usually comprises above quality factors and contains at least one more category between “good” and “ungradable”/“poor” for human graders^9^. Binary automated RIQA algorithms that classify an image as “good” or “poor”^8,10^ seems not applicable in real-world clinical environment. Because RIQA is essentially a regression problem^11^ and a binary classifier is an inflexible approach. In multi-class RIQA approaches, recent works^12,13^ introduced a third quality label named as “ambiguous” or “usable” and employed convolutional neural network (CNN) as quality classifiers. Earlier studies^14,15^ aimed to design quantitative metrics for segmented retinal vessels from a retinal image. We also acknowledge the visibility of retinal vessels is a great quality indicator as stated in a standard recommendation^9^. Structural information of the retinal vessels is one of discriminative features in many RIQA methods^16–19^. A fundamental assumption behind all these vessel-based approaches is that poor vessel segmentation is caused by poor image quality. We could evaluate structural characteristics of vessel segmentation so as to assess retinal image quality.

The main concern of vessel-based approaches is the reliability of automated vessel segmentation method in cross-dataset evaluation. For example, B-COSFIRE^20^ is a matched filter approach and might require parameter adjustment for testing on different datasets. It is unlikely to get real-time and stable vessel prediction. Recent machine learning advances applied CNN to retinal vessel segmentation and demonstrated state-of-the-art performance on public database^21^. The learning based approach demands no parameter adjustment in the test stage. Nevertheless, segmentation performance on large-scale database is still unknown due to lack of massive manual-labeled vessels. If a CNN model is applicable to real-time accurate vessel prediction, we can utilize it on previous vessel-based RIQA methods. Among these studies, Hunter et al.^14^ proposed a vascular metric to indicate clarity of vessels within the macula region. Fleming et al.^17^ extended vessel clarity to entire field of view (FOV). They extracted a set of structural and statistical features from a square box centered at strong edge response of a Gaussian filtered image. Then, they classified each box as clear or blur. Other works extracted vessel features from segmented vessel images locally^16,18^ or globally^15,19^. They proposed local and global vessel density^15,16,18^ to present a ratio of vessel area over the area of image patch or whole image respectively. In addition to vessel density, researchers also devised vessel shape and complexity descriptors^19^ as image features to identify images with inadequate quality.

The hand-crafted vascular features are frequently used in RIQA systems before CNN classifiers. From our perspective, the vascular features are more explainable than CNN features even though there is a popular heatmap technique to interpret model’s decision on image quality^22^. We extend previous vessel-based RIQA methods and propose fractal dimension (FD) of retinal vessels as a global vascular feature. Vascular fractal is highly associated with retinal image quality^23^ meeting basic assumption of vessel-based RIQA. FD can measure vascular geometrical complexity since the branching patterns of retinal vasculature is a fractal, a geometrical pattern whose parts resemble the whole^24^. A larger FD represents a more complex pattern. We obtained the highest FD value of 1.7 from a high-quality normal fundus image. On the contrary, ungradable images are assumed to have small FD because of poor vessel segmentation result. The FD value is close to 0 if there is only a few segmented vessels. Besides, a previous clinical study^25^ shows the FD value is associated with various diseases, such as DR. Less visible vascular structure is commonly seen in proliferative DR (the most severe grade of DR). The major limitation of proposed vascular fractal based RIQA is the inability to classify images with decreased vessel density or inadequate field definition.

In this study, we propose the FD of retinal vasculature as a novel RIQA indicator. We employ our improved CNN model to segment retinal vessels of a standardized image and use box counting method^26^ to calculate FD of each vessel segmentation. Vessel segmentation model is trained on our finer vessel annotation dataset RETA^27^. An image will be considered as poor quality if its FD is less than an empirically quality threshold. Experimental results on four public image quality datasets demonstrate our method is applicable and explainable for the RIQA task. The FD value working as a one-dimension quality indicator is validated to be a robust metric and the estimated threshold of inadequate image quality is between 1.45 and 1.50. Healthcare provider is able to set a flexible quality threshold in different application scenarios.

## Results

### Data acquisition

Four publicly available RIQA datasets are studied in this paper. They are HRF-quality^28^, DRIMDB^18^, EyeQ^13^ and DeepDRiD^29^. HRF-quality database contains 18 image pairs captured with a 45$^\circ$ FOV and different acquisition settings. For each pair, only one image shows adequate quality and both images are from the same subject. DRIMDB is a frequently used public dataset for RIQA. There are 125 images of good quality, 69 images of bad quality and 22 images belonging to outlier. Outlier refers to nonretinal images such as anterior segment images and generic natural images. All good images are macula or OD centered images. Most images are stored in JPG format with 760 $\times$ 570 pixels. EyeQ consists of 12,543 training images and 16,249 test images labeled as “Good”, “Usable” and “Reject”. All images are from EyePACS. “Good” and “Reject” denote high and low image quality. “Usable” images show quality issues, such as artefacts, blur, overexposed, underexposed and uneven illumination, but they still show visible retinal anatomical structures and lesions. Apart from overall image quality label, DeepDRiD provides detailed quality scores (5-level grading scale) in terms of artifacts, clarity and field definition. It comprises 1200 images for training and 400 images for validation. However, we recommend readers to carefully deal with the provided noisy labels. Furthermore, the DR labels are available in both EyeQ and DeepDRiD. A higher DR level is associated with decreased geometric complexity of the retinal vasculature. It would be of special interest to analyze FD within different DR levels.

### Qualitative evaluation

**Figure 1:**
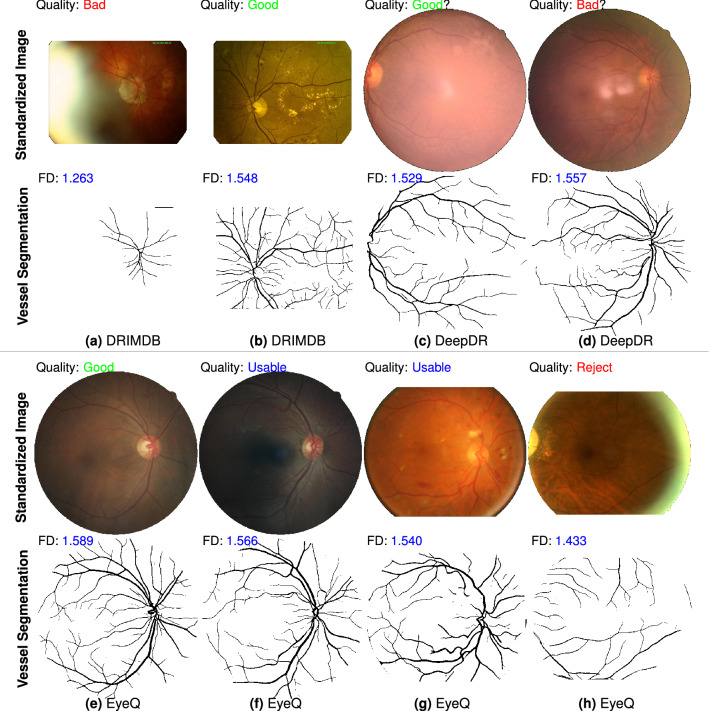
Qualitative comparison of proposed vascular fractal based method for automated image quality assessment. There are 8 standardized fundus images from DRIMDB, DeepDR and EyeQ datasets (image credit: (**a**) “drimdb_bad (17)”, (**b**) “drimdb_good (19)”, (**c**) “280_l1”, (**d**) “214_r2”, (**e**) “8715_right”, (**f**) “34444_right”, (**g**) “31264_left”, (**h**) “39148_left”). Quality labels given by human graders are on the top-left corner of each image. Vessel segmentation image (predicted by our segmentation model) and measured FD are displayed at the bottom of each color image. We are skeptical about the correctness of quality labels of (**c**,**d**). The vessel segmentation and FD results may visually support our suspect. The third row shows 4 standardized fundus images labeled as (**d**) good quality, (**e**) usable but show uneven illumination, (**f**) usable but with partial blur and (**g**) bad quality due to inadequate field definition and over-exposure. Vessel segmentation results of (**a**,**h**) show our model is sensitive to over-exposure issue. An image with higher quality generally has a larger FD.

We qualitatively compare performance of the proposed FD metric on RIQA task. Figure 1 shows standardized images from three datasets (DRIMDB, DeepDRiD and EyeQ), reference quality labels, segmented vessel images predicted by our CNN-powered segmentation model and measured FD values. The standardized color images in Fig. 1a,b are from DRIMDB dataset. Figure 1a is an over-exposed fundus image characterized by milky-white layer from image periphery to center. A small FD is calculated from its small-scale visible vessels. Figure 1c,d are two images with probably incorrect quality labels because they are inconsistent with the calculated FD of vessel vasculature. One obvious issue is the inadequate field definition of color image in Fig. 1c. Figure 1e–h displays four retinal images from EyeQ database but they are misclassified by MCF-Net^13^. The measured FD values based on our vessel predictions can properly indicate the image quality. The smallest FD is 1.433 denoting insufficient overall quality of the “Reject” image in Fig. 1h. Our vessel segmentation model works well in RIQA task distinguishing bad quality images from good quality images. To be specific, our segmentation model is sensitive to images with bad quality like the over-exposure issue. It also produces a finer vessel segmentation image with almost all visible tiny vessels (e.g. Fig. 1b). To conclude, Fig. 1 illustrates the computed FD values on vessel segmentation images are closely related with given image quality labels.

### Quantitative evaluation

#### HRF-quality dataset

**Figure 2:**
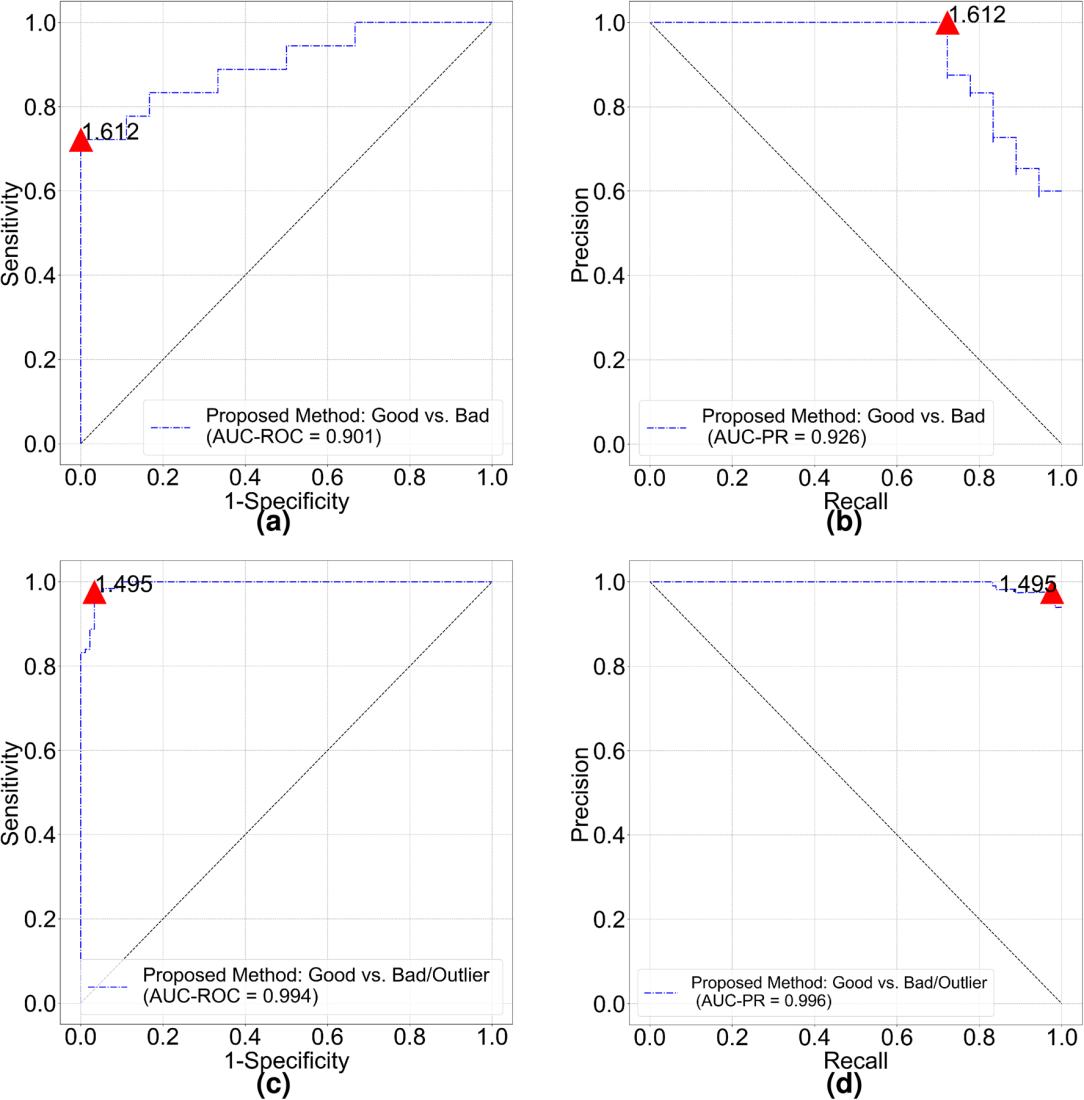
Classification performance of proposed vascular fractal based method. (**a**) ROC curve and (**b**) PR curve for HRF-quality database. (**c**) ROC curve and (**d**) PR curve for DRIMDB image set. Red triangle on each curve indicates optimal cut off point and the number is corresponding classification threshold.

The mean FD of “good” and “bad” quality groups are 1.615 (95% confidence interval [CI] 1.603–1.627) and 1.559 (95% CI 1.539–1.579) respectively. The FD of good quality is significantly larger than FD of bad quality (*p* = 4e−05). Figure 2 shows receiver operating characteristic (ROC) and precision recall (PR) curves for image quality classification. The FD threshold for quality classification depends on optimal cut-off values of the ROC and PR curves. The overall accuracy of quality classification is 80.56% if FD threshold is set to 1.60. The accuracy is relatively low because most of bad quality images only show decreased sharpness (out of focus) compared with good quality images. From vessel segmentation results, we notice our model could still segment blood vessels quite well from bad quality images. In a subset of HRF-quality that only contains decreased sharpness images and corresponding good quality images, the mean FD of “good” quality and “bad” quality groups turn to 1.614 (95% CI 1.600–1.629) and 1.575 (95% CI 1.563–1.587) respectively (*p* = 3e−4). The accuracy drops to 76.67% with the same classification threshold. That means our segmentation model cannot identify images with decreased sharpness in HRF-quality. Nonetheless, it implicitly shows advanced generalization capability of our vessel segmentation model.

#### DRIMDB dataset

The mean FD of “good”, “bad” and “outlier” groups are 1.565 (95% CI 1.560–1.571), 1.057 (95% CI 1.008–1.105) and 1.303 (95% CI 1.203–1.403) respectively. All the groups are significantly different from each other ($p<0.001$). For a binary quality classification (“good” vs. “bad”/“outlier”) model, cut-off values of ROC (Fig. 2c) and PR curves (Fig. 2d) are both 1.495. Area under the ROC curve (AUC-ROC) and Area under the PR curve (AUC-PR) are 0.994 and 0.996, respectively. Classification accuracy of good quality images and all images are 97.60% (122/125) and 97.22% (209/216) respectively.

#### DeepDRiD dataset

**Figure 3:**
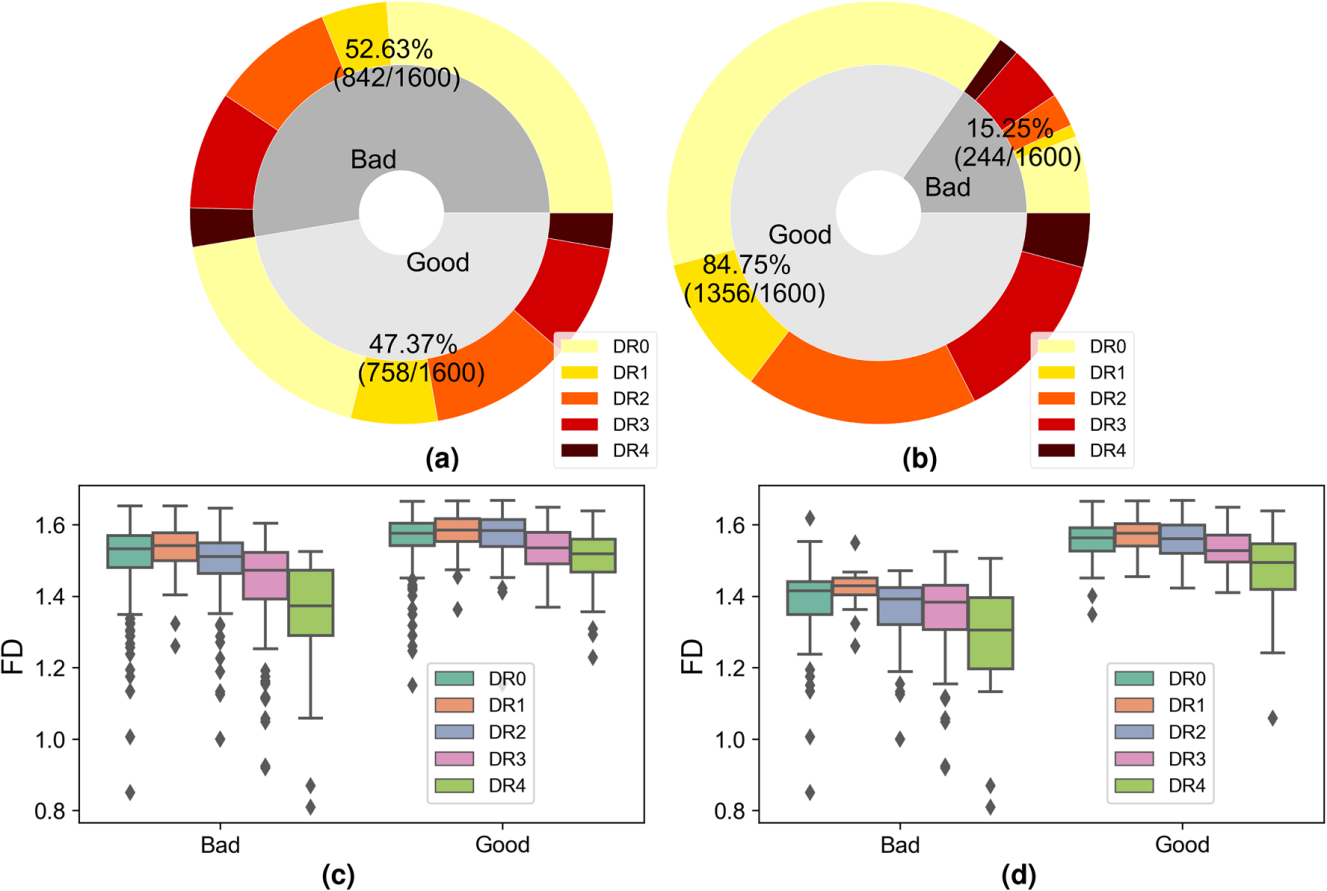
Top row: image distribution with respect to image quality and DR level in DeepDRiD. Pie plots of (**a**) original dataset and (**b**) label-revised dataset. For the severity of DR, a larger number refers to the higher risk of vision threatening. DR0 refers to normal and DR4 is the sight-threatening level. Bottom row: statistical results of FD in different image quality groups and DR levels. Box plots of (**c**) original and (**d**) label-revised datasets. In (**d**), there are some outlier points of good quality images in DR0 and DR4. They refer to images with decreased vessel density. In bad quality group of (**d**), outlier points with large FD values are observed and these images are labeled as low quality because of inadequate field definition. However, there are clear visibility of macular vessels in images resulting into unexpected high FD.

RIQA is a subjective task for human graders and noisy labels can be easily introduced in the process of large-scale database construction due to inter-observer and intra-observer variability. The proposed method can also work as an effective approach for erroneous label identification and validation. Figure 3 (a) shows sample distribution of bad and good quality groups. We visualize measured FD values with respect to image quality and DR levels in Fig. 3c. There are lots of outlier points in both good and bad quality group. A basic assumption of our method is that a small FD is more likely to be from a bad quality image. After double-checking color retinal images and corresponding FD values, we modify original quality labels and obtain a revised label distribution shown in Fig. 3b. Mislabeled images with high FD values are pretty easy to identify. We category images with quality issues but still gradable as good quality. Therefore, a clear classification borderline between good and bad quality is obtained.

In the original dataset, the mean FD of good and bad quality groups are 1.559 (95% CI 1.555–1.564) and 1.488 (95% CI 1.481–1.496). AUC-ROC and AUC-PR are 0.74 and 0.71. An optimal quality threshold for original dataset is 1.548 obtained from the ROC curve. Filtering out bad quality images below this threshold, we get a binary classification accuracy of 67.13%. The issue of low classification accuracy is also argued in the DeepDRiD challenge report^29^. Their results are evaluated on private labels of 400 test images. Whereas in the label-revised dataset, the mean FD of good and bad quality groups become 1.551 (95% CI 1.548–1.554) and 1.351 (95% CI 1.329–1.372). AUC-ROC and AUC-PR become 0.970 and 0.993. The quality threshold is set to 1.450 based on the cut-off value of the PR curve and it is also close to the cut-off value 1.471 of ROC curve. The classification accuracy of new DeepDRiD dataset rises to 95.88%.

**Figure 4:**
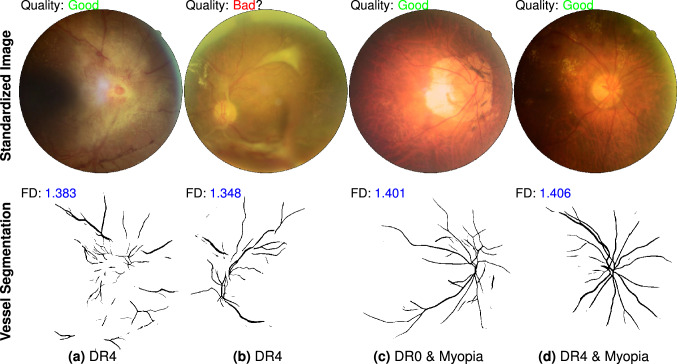
Failed examples caused by decreased vessel density in DeepDRiD dataset. Top row shows 4 pathological images with sufficient image quality and the second row displays corresponding vessel segmentation images predicted by our retinal vessel segmentation model. Misclassification is owing to the presence of only a few visible vessels within the FOV. The file names of color images are (**a**) “51_r1”, (**b**) “309_l2”, (**c**) “67_r2” and (**d**) “265_r1”.

We investigate the outlier points in Fig. 3d and their influence for RIQA. High FD in bad quality group and low FD in good quality group are two unacceptable cases that will degrade classification performance of RIQA. In the bad quality group, we find 11 images with inadequate field definition but a large FD (> 1.50). A retinal image with adequate field definition was defined as one that shows the OD and at least two OD diameters of visible area around the fovea^9^. In the binary classification task of good/bad quality images, we aim to pass good quality images for ARIA systems as many as possible. False positive rate (FPR) caused by inadequate field definition is estimated as 11/244 = 4.5% if we set 1.50 as the classification threshold. In the good quality group, small FD values are observed from those images with decreased vessel density. For the DR4 category, there are 25 and 67 samples in bad quality and good quality groups respectively. The mean FD of good and bad quality groups are 1.472 (95% CI 1.447–1.497) and 1.283 (95% CI 1.217–1.350). There exists significant difference between them (*p* = 6e−9). Only a small part of retinal images will display significant decrease of vessel density in the stage of DR4, pathological myopia or other conditions. Figure 4 shows representative images with sufficient quality from DeepDRiD. They all have small FD values due to obscured visibility of retinal vessels. To estimate the false negative rate (FNR) as a consequence of decreased vessel density in DR4, we double-check all 37 good quality images whose FD are less than 1.50. There are 23 images actually showing inadequate image quality because they have borderline quality. DeepDRiD contains only binary quality labels unlike trinary labels of EyeQ. They can be correctly classified when applying our method to the quality improved images. Therefore, if we set the quality threshold to 1.50, the FNR of DR4 images will be 14/67 = 20.9% (67 is the number of all good quality images in DR4 group). Certainly, the FNR will be smaller if we decrease the threshold.

#### EyeQ dataset

**Figure 5:**
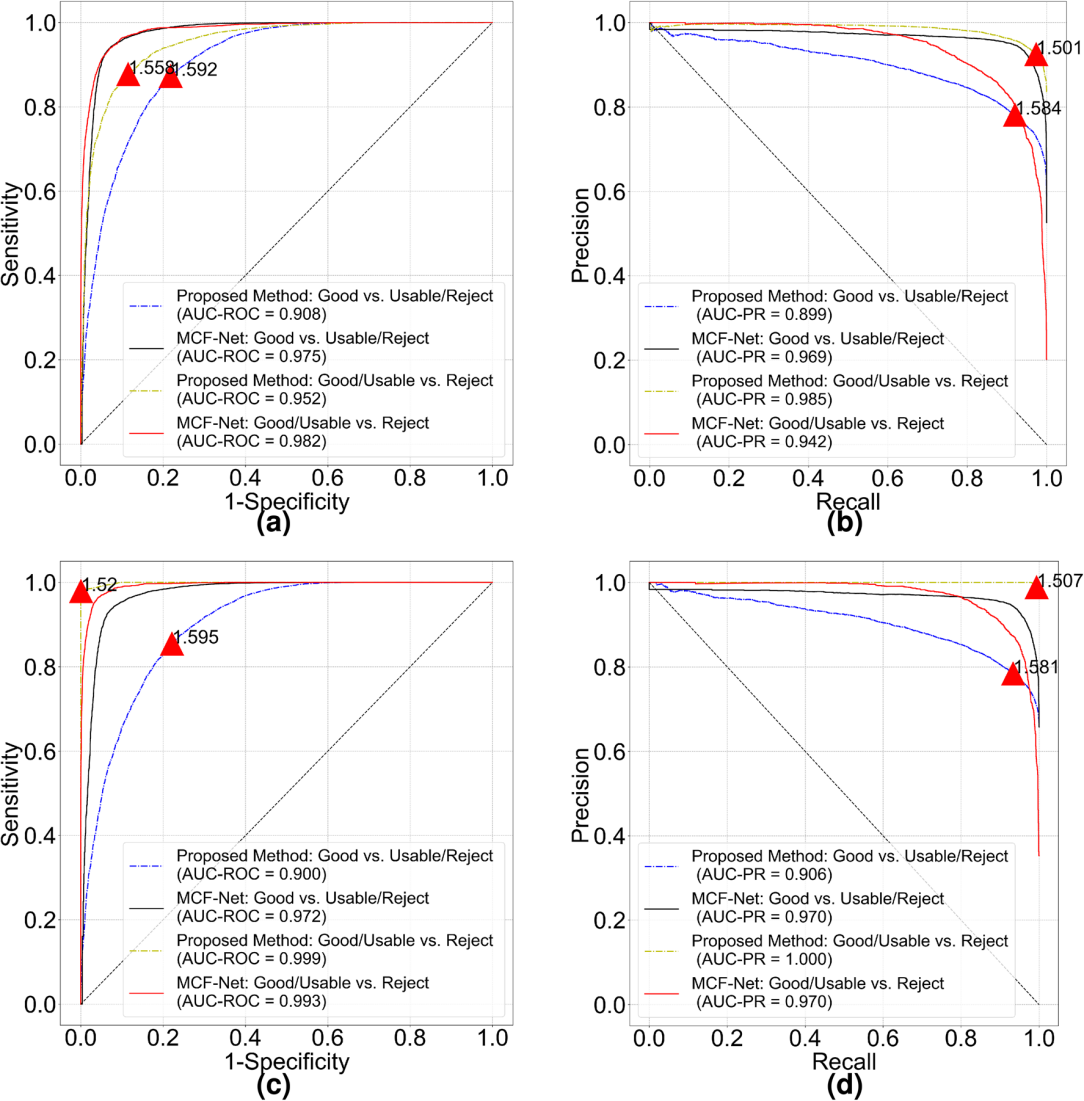
Performance comparison of the MCF-Net and proposed methods for classifying “Good” and “Reject” images in EyeQ test set. (**a**) ROC and (**b**) PR curves are measured on the original labels from human graders. We show revised ROC and PR curves in (**c**,**d**) to indicate classification performance after removing potential incorrect labels. Red triangle and numerical digit on each curve indicate the optimal cut off point and FD threshold of the proposed method.

For all images in EyeQ test set, the mean FD of “Good”, “Usable” and “Reject” groups are 1.620 (95% CI 1.620–1.621), 1.570 (95% CI 1.569–1.572) and 1.385 (95% CI 1.377–1.393), respectively. T-test results show that all the quality groups are significantly different from each other ($p<0.001$). We quantitatively compare RIQA performance of MCF-Net and proposed method in Fig. 5a,b. The FD threshold of “Good” quality image classification is close to 1.60 and the AUC-ROC of our method is 0.908. For “Reject” image classification, MCF-Net and proposed method achieve much higher AUC-ROC. Our method shows better performance in terms of the AUC-PR. The ideal threshold of proposed method for “Reject” image classification is 1.501 obtained from the PR curve due to imbalanced class distribution. It seems easier to separate “Reject” images than “Good” images in EyeQ test set. Because there is a fuzzy borderline between the “Good” and “Usable” images. Finally, we study the classification accuracy of proposed method in a multi-class task. Table 1 is the results at different quality thresholds. We feel special interested in the low classification accuracy of “Reject” images at 1.50 quality threshold. Based on our understanding of the quality grading scale described in EyeQ paper^13^, these images with FD above 1.50 can actually be categorized into “Usable”.

**Table 1:** Multi-class classification accuracy of EyeQ test set using proposed method.

Threshold	Good (%)	Usable (%)	Reject (%)
1.45	100	97.98	19.53
1.50	99.95	90.15	38.11
1.55	98.29	73.33	56.21
1.58	93.64	49.46	94.53
1.60	81.04	26.63	97.83

For each quality class (“Good”, “Usable” or “Reject”), a quality threshold is applied to filtering out negative images and compute corresponding accuracy.

Following our noisy label identification method in DeepDRiD dataset, we identify potential erroneous labels of EyeQ dataset. After excluding 1138 images with uncertain labels, we obtain the revised ROC and PR curves shown in Fig. 5c,d. The proposed method obtains much higher AUC-ROC and AUC-PR of 0.9991 ($\approx 1.00$) and 0.9998 ($\approx 1.00$) respectively for the “Reject” image classification. The list of images with questionable label is available at attached supplementary document.

We also evaluate the RIQA performance of proposed method on EyeQ training set. The mean FD of “Good”, “Usable” and “Reject” groups are 1.621 (95% CI 1.620–1.621), 1.565 (95% CI 1.563–1.567) and 1.371 (95% CI 1.362–1.380). The AUC-ROC and AUC-PR of “Reject” image classification are 0.97 and 0.99. As for “Good” image classification, the AUC-ROC and AUC-PR of our method are 0.94 and 0.96, respectively. Our model shows better classification performance of the training set than test set. In conclusion, the experimental results of EyeQ dataset demonstrate our proposed method is effective for retinal image quality classification.

**Figure 6:**
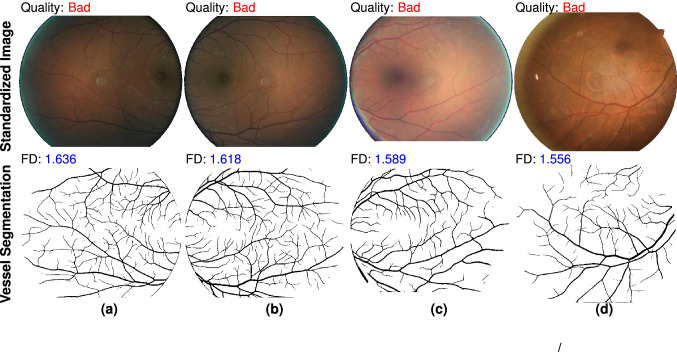
Inability of the proposed method to classify images with inadequate field definition. Top row shows four color fundus images from EyeQ training set and the second row are corresponding vessel segmentation images. OD is not visible in the FOV. A big FD value is obtained due to clear visibility of macular vessels. Original file names of the color fundus images are (**a**) “24871_left”, (**b**) “24871_right, (**c**) “34207_right” and (**d**) “38160_right”.

We further study the FPR and FNR attributable to inadequate field definition and decreased vessel density, respectively. The study samples along with quality labels are from entire EyeQ image set including the training and test sets. Figure 6 shows 4 color fundus images with inadequate field definition. The OD regions are invisible in FOV. Our vessel segmentation model is able to segment visible macular vessels and the FD values are large than our recommend threshold 1.50. Nonetheless, inadequate field definition is one important aspect in standard grading scale^9^. So, this result seems not acceptable for a standard screening programme. We identify 231 images having this issue in EyeQ. Thus, in a total of 5540 “Reject” images, the FPR of our method can be calculated as 231/5540 = 4.17%. To deal with decreased vessel density, we search images with FD above 1.50 in both the “Good” and “Usable” groups. In total, 53 candidate images are checked and categorized into 2 classes, decreased vessel density and inadequate image quality. Only 23 images are graded as decreased vessel density (4 images from “Good” and 19 images from “Usable”). The rest images are in the “Usable” quality group. We believe it is the quality issues of these images that makes the propose method failed. Table 2 is the confusion matrix for DR4 images classification. There are 13 images showing sufficient image quality but mislabeled by human graders. The classification accuracy could be 96.45% when the quality threshold is 1.50. We calculate the FNR of DR4 images classification as 23/(272 + 23) = 7.80%. That means there is about 7.80% DR4 images will be wrongly classified by reason of decreased vessel density. The source data is available in the "Supplementary data". In a word, our method shows weakness to classify images with inadequate field definition and decreased vessel density.

**Table 2:** Confusion matrix of image quality classification for DR4 images of EyeQ dataset using our approach.

	Predicted: good/usable	Predicted: reject
Labeled: good/usable	272	23
Labeled: reject	13	340

The quality threshold is set to 1.50 and there are 13 images mislabeled as “Reject” quality by human graders. Our method is unable to correctly classify 23 images with decreased vessel density.

## Discussion

The first essential step of a ARIA system is identification and exclusion of ungradable fundus images. It acquires images of sufficient quality to be assessed ensuring reliable medical diagnosis. In such systems, RIQA module plays a significant role in human-machine interaction. For an image of inadequate quality, one common workflow is to retake another image that meets the minimum image quality standards of RIQA. However, frequent rejections will discourage humans if there is no convincing explanation of the low quality decision. In addition, binary classification of image quality such as “good” or “poor” seems not the optimal choice for real-world RIQA applications. A continuous quality scale is preferable^11^. Therefore, in the interest of applicability, a RIQA algorithm should be explainable and grades image quality on a continuous scale.

Explainability of a RIQA algorithm could help human understand its rejection decision. The proposed vascular fractal based method and many other vessel-based approaches are intrinsically explainable since poor vessel segmentation results from unacceptable image quality. An explainable RIQA is significant in human-machine interaction. Unfortunately, the model interpretation of CNN-based RIQA method is still an open resolved topic in community. We believe visual hint directly from segmented vessel image is better than merely one quality tag. A suitable description of the reason of poor-quality image could help technicians retake a fundus image with improved quality.

Grading of image quality should be on a continuous scale. Healthcare providers can freely define the number of quality classes and image quality standards based on local available ophthalmoic apparatus (tabletop, handheld, or smartphone-based fundus cameras). In this case, a vessel-based RIQA method is a better option for ARIA systems. For machine learning based RIQA methods^12,13^, it is expensive and time-consuming to rebuild image quality training set for new quality standards. Moreover, noisy labels can be easily introduced by annotator variability. One example is DeepDRiD dataset in this study. In most cases, there will be three image quality grades, for instance, “Good”, “Usable” and “Reject”. From our perspective, RIQA had better be a regression task rather than a classification one. Karlsson et al.^11^ proposed an image quality scale between 0 to 1. In our approach, image quality ranges from 0 to 1.7. Users can flexibly select thresholds for different quality classes using the continuous grading scale. For fundus images captured by handheld and smartphone-based fundus cameras^30^, a small threshold must be considered because these images usually have small FOV and image resolution.

Selection of a proper vessel segmentation model and quality threshold for RIQA depends on model generalizability and image quality grading scale. Large-scale cross-dataset validation of vessel segmentation models is required for choosing a robust segmentation model. To the best of our knowledge, this study is the first work to illustrate automated vessel segmentation performance in a large-scale database. The quality threshold of inadequate quality images relies on defined quality standards and the FOV shape of fundus images. In DRIMDB database, we observe that there is almost rectangular shape of the FOV region and it has impact on measure FD (a relatively small value). The best quality thresholds for inadequate quality of DRIMDB, DeepDRiD and EyeQ datasets are 1.495, 1.450 and 1.507, respectively. Thus, we suggest that the threshold of poor quality images can be set to [1.45, 1.50]. High quality images generally have the FD values between 1.60 and 1.70. Users are able to flexibly select a quality threshold.

Last but not least, the limitation of proposed vascular fractal based method is a lack of capability to classify images of inadequate field definition and decreased vessel density. We have showed several examples in Figs. 4 and 6. In EyeQ dataset, the FPR of rejected images and FNR of DR4 images are 4.17% and 7.80%, respectively. Notwithstanding, there are some solutions to overcome these disadvantages of proposed method and improve the RIQA performance. To decrease the FPR caused by inadequate field definition, we could employ another image segmentation module to locate OD region^9,31^. If the OD is absent from an image, the RIQA had better reject this image. To reduce the FNR caused by decreased vessel density, one possible solution is taking into consideration the DR grades while making image quality decisions. If one image is graded as DR4, the RIQA needs to double-check its vessel segmentation and corresponding FD. In addition to DR4, decline in vessel density is also commonly seen in pathological myopia and other diseases. We suggest healthcare providers to evaluate the influence of FNR on the overall performance of ARIA systems.

## Conclusion

In this study, we propose an easy, effective and explainable novel metric, fractal dimension of retinal vasculature, for automated image quality assessment in ARIA systems. This metric is calculated on retinal vessels extracted by our advanced CNN-based vessel segmentation model. We use box counting method to estimate the vascular fractals and apply it as the single dimension feature to evaluate retinal image quality. Experiments on large-scale public datasets verify fractal dimension measured on our vessel segmentation images is a simple and effective image quality indicator for automated fundus screening systems. The quality threshold of images having adequate overall quality can be flexible depending on different application environment. A larger threshold generally requires the uploaded images with higher quality.

## Methods

### Image standardization

**Figure 7:**
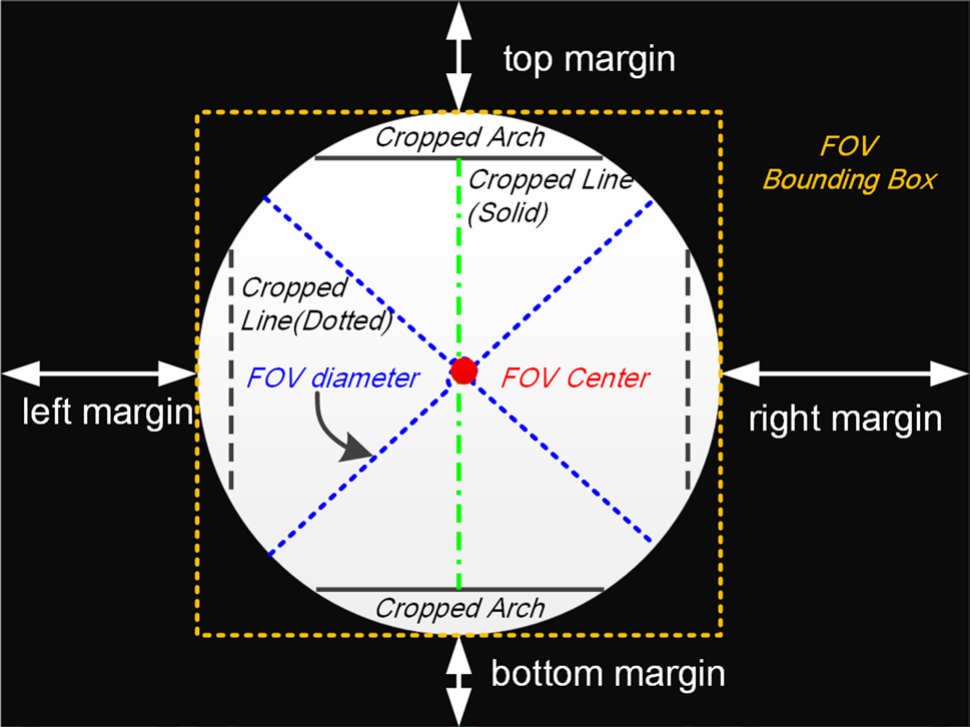
Typical structural composition of a fundus image. FOV is a circle shaped region highlighted by a yellow bounding box. The length of FOV bounding box equals to FOV diameter. The colorized FOV region is surrounded by black pixels. Ideally, there is no circular segment cropped from the FOV. The FOV center is the image center. The left and right margins have the same length. So are the top and and bottom margins.

The optical design of fundus camera is based on the indirect ophthalmoscope and uses a doughnut shaped aperture^32^. Reflected light from retina passes through the hole of doughnut and forms a circular shaped fundus image. A angle of view refers to optical acceptance angle of camera lens. A fundus camera typically can view 30$^\circ$–60$^\circ$ of retinal area (also called FOV). Fundus images taken by different cameras and operators may vary in image resolution, dimension, the FOV shape and size. Figure 7 is a simple illustration of a fundus image. A circular FOV region is surrounded by black image background. Sometimes small circular segments will be invisible mostly occurring at the top and bottom of FOV. In this case, height and width of the FOV bounding box are different.

Image standardization process aims to remove black pixels outside the FOV region and resize it into a fixed dimension. This is a certain image preprocessing step because there is a fixed input size of vessel segmentation model. Moreover, a proper FD measurement is in aligned fundus images. In the study of Cheung et al.^25^, an image is aligned based on OD diameter (ODD) and the FD measured area is the region from 0.5 to 2.0 ODD away from disc boundary. However, for the RIQA application, we extend FD measured area to entire FOV since we would like to quantify global instead of regional image quality issues. The RIQA system requires a global FD estimation of retinal vessel structures. Our image standardization includes four steps as follows:

1. Extract a binary FOV mask from the original color image;
2. Locate a square bounding box. The FOV mask is an inscribed circular region of this box. Any missing circular segment will be filled with black pixels;
3. Crop original color image using top-left and bottom-right coordinates of bounding box;
4. Resize the cropped color image into a fixed dimension (in this paper, it is 1024 $\times$ 1024).

### Automated vessel segmentation

**Figure 8:**
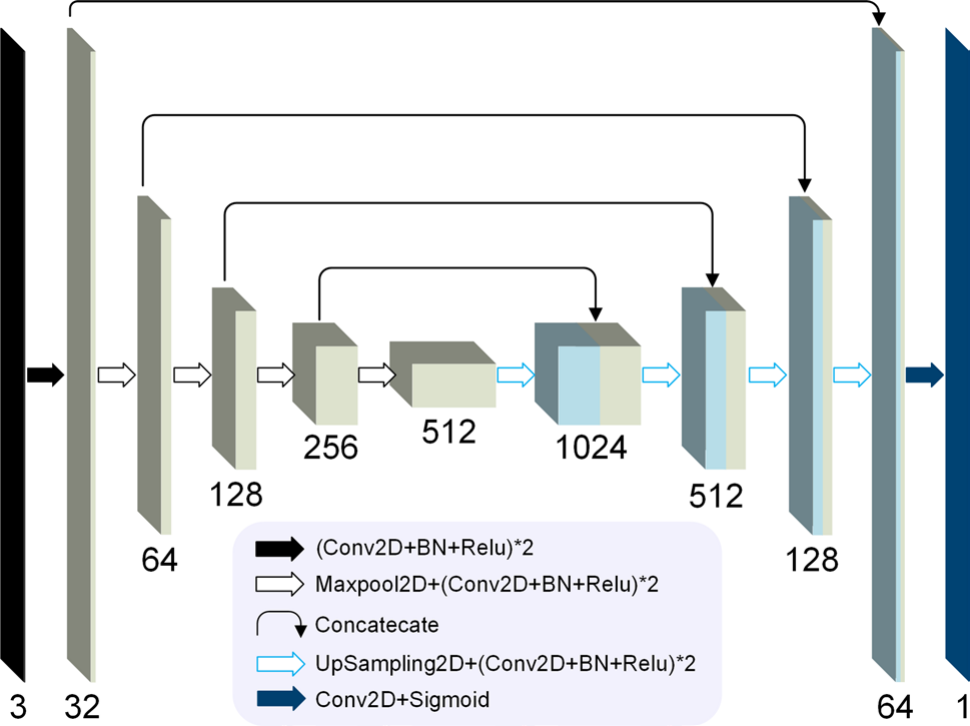
The architecture of our vessel segmentation model. It is an encoder–decoder convolutional neural network. Model input is a 1024 $\times$ 1024 color retinal image and model output is single channel 1024 $\times$ 1024 vessel prediction image. Conv2D is 2D convolutonal layer with kernel size of (3,3). Padding parameter of Conv2D is set to “same” in order to output the same dimension of the input. BN refers to BatchNormalization layer. Output channels of encoding blocks are 32, 64, 128, 256 and 512 respectively while output channels of decoding blocks are 512, 256, 128, 64 and 32.

Let $I_{CF}$ be a two-dimension color fundus image and $I_{GT}$ be corresponding vessel image manually labeled by human annotators. Each pixel in an image has an Euclidean coordinate $(x,y), x,y\in {\mathbb {Z}}^+, x \le M, y \le N$, where *M* and *N* are image height and width. Vessel segmentation task is a pixel to pixel projection problem using a mapping function $\Phi (I_{CF}; \Theta ) \rightarrow I_{GT}$, where $\Theta$ denotes a weight set. We design a encoder-decoder CNN model to learn $\Phi$ between $I_{CF}$ and $I_{GT}$. $\Theta$ is iteratively updated by minimizing a binary cross entropy loss function *L* defined as

1 $$\begin{aligned} \small L = \frac{1}{M \cdot N}\sum _{x=1,y=1}^{M,N}-I_{GT}^{x,y}\cdot log\left( I_{PR}^{x,y}\right) -\left( 1-I_{GT}^{x,y}\right) \cdot log\left( 1-I_{PR}^{x,y}\right) \end{aligned}$$

where $I_{PR}=\Phi (I_{CF}; {\hat{\Theta }})$ is vessel prediction image using learned weight ${\hat{\Theta }}$. $I_{GT}^{x,y}\in \{0,1\}$ and $I_{PR}^{x,y}\in [0,1]$. In our model, $M=1024,N=1024$. Encoder module has five feature encoding blocks and decoder module has four decoding blocks. 3 $\times$ 3 convolutional filter is used to extract image features. Figure 8 shows model architecture of our vessel segmentation method. It is trained on 1600 images augmented from 54 images of our newly annotated dataset RETA^27^. Augmentation methods include image rotation and flipping. $\Theta$ is optimized by Adam optimization algorithm with an initial learning rate of 2e−4. Training epoch is set to 6.

### Fractal dimension analysis

A commonly adopted approach for FD estimation in Euclidean space is box-counting dimension^26^. Given a binarized vessel prediction $I_{B}$, overlay an evenly spaced grid of boxes of size $\varepsilon$ and count the number of boxes required to cover all vessel pixels. Figure 8b–f shows there are finer details of retinal vasculature from the covering by decreasing $\varepsilon$. Let $N(\varepsilon )$ be box number as a function of $\varepsilon$, the box-counting dimension of $I_{B}$ is estimated as the exponent of a power law

2 $$\begin{aligned} \small FD(I_{B}) = \lim _{\varepsilon \rightarrow 0} \frac{\log N(\varepsilon )}{\log 1/\varepsilon } \end{aligned}$$

**Figure 9:**
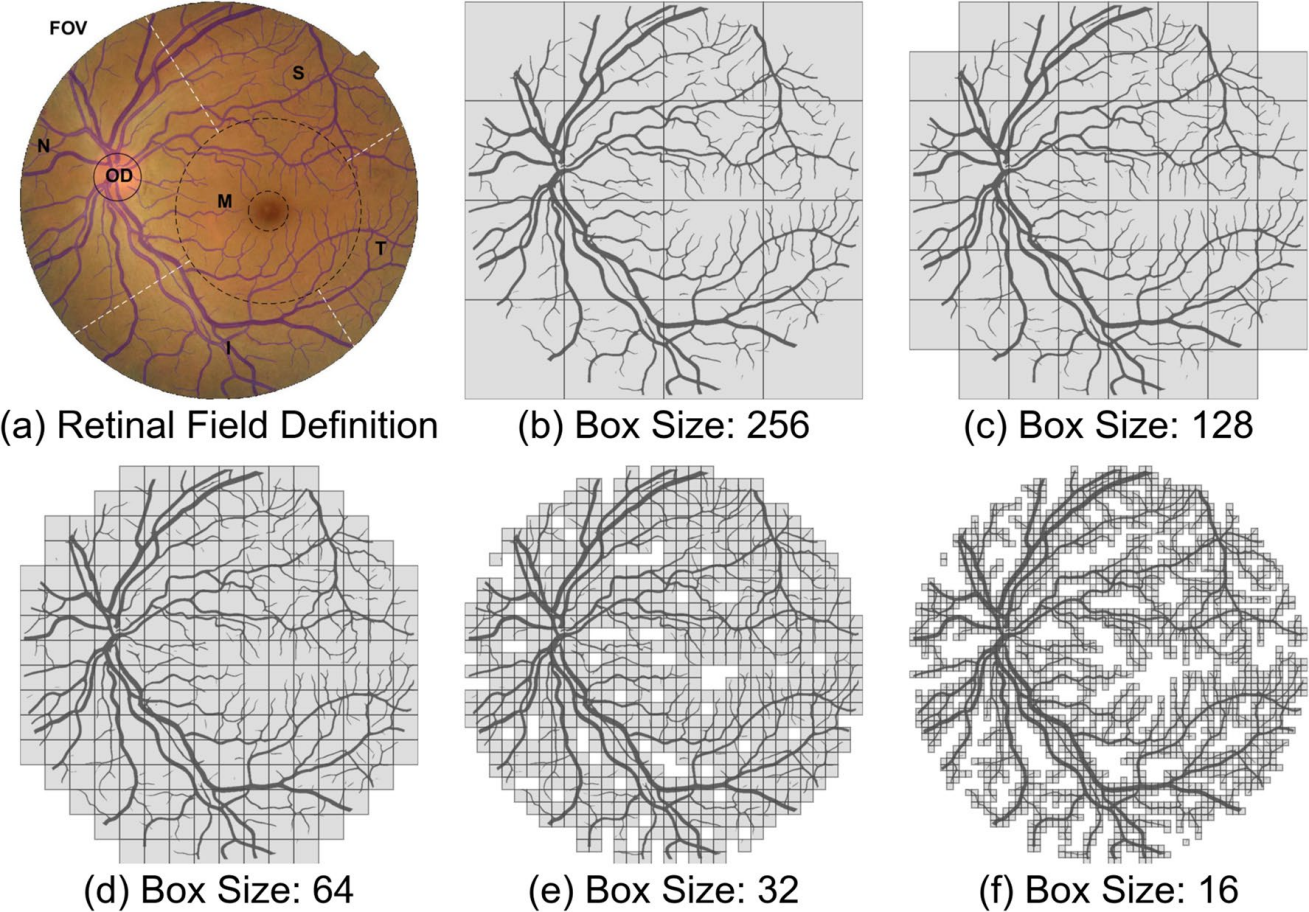
An illustration of fractal dimension analysis with application to fundus image. (**a**) Six retinal fields defined in a color fundus image overlaid with vessels. This color retinal image is from EyeQ test set (“26088_left.jpj”). From (**b**–**f**), box counting process by covering five boxes of different sizes on vessel segmentation of (**a**).

Figure 9a divides the FOV region into six sub-regions. The overlaid blue vessel segmentation is predicted by our vessel segmentation model. Here, M denotes macula region. Taking *d* as the distance from OD center to M center, the radius of M is estimated as $0.6\times d$. A smaller dotted circle in M represents fovea region. Inferior(I), superior(S), nasal(N), and temporal (T) are four quadrants with M in the center. For a retinal field set $FOV = \{OD,M,N,S,T,I\}$, we manually remove one of visible retinal fields from vessel segmentation image, the calculated FD of remaining visible vessel structure is in Table 3. The FD value of FOV region is 1.696. Any removed sub-region within the FOV will contribute to a smaller FD. We get the smallest FD when retinal vessel is only visible inside the OD. Images with inadequate field definition show small FD values and they can be easily rejected by our approach. Table 3 verifies the FD is a robust metric for quantifying visible retinal vessels. Any image quality problem will lead to decreased visibility of vessels and subsequently result in a smaller FD. The FD value actually depends on the visibility of retinal vessels.

**Table 3:** Measured FD with respect to visible retinal fields.

Visible field	FD	Visible field	FD
FOV	1.696	$\varnothing$	NaN
OD	0.985	FOV$\setminus$OD	1.692
M	1.394	FOV$\setminus$M	1.645
N	1.545	FOV$\setminus$N	1.620
S	1.446	FOV$\setminus$S	1.654
T	1.308	FOV$\setminus$T	1.676
I	1.397	FOV$\setminus$I	1.660

“NaN” denotes FD cannot be calculated.

### Statistic analysis

We visualize the performance of binary classification model with the ROC and PR curves. The ROC curve plots true positive rate (*TPR*) versus false positive rate (*FPR*) and the PR curve plots precision *P* versus recall *R* at different classification thresholds. The ROC curve is appropriate for balanced class distribution whereas the PR curve is suitable for imbalanced datasets. The higher the AUC-ROC or AUC-PR, the better the classification model is. The calculations of *TPR*, *FPR*, *P* and *R* are

3 $$\begin{aligned} TPR = \frac{TP}{TP+FN}, FPR = \frac{FP}{FP+TN}, P = \frac{TP}{TP+FP}, R = \frac{TP}{TP+FN} \end{aligned}$$

where true positive (TP) and false negative (FN) denote the number of sufficient quality images that are correctly and wrongly classified by quality classifier respectively. Similarly, true negative (TN) and false positive (FP) represent the number of correctly and wrongly classified insufficient quality images.

Besides, independent sample t-test is used to analyze the mean comparison of two independent groups with respect to the FD. A *p* value smaller than 0.001 is considered statistically significant. We examine if the mean of FD is statistically different between sufficient quality and insufficient quality groups.

## Supplementary Information

Supplementary Information 1.

Supplementary Information 2.

## Data Availability

The public RIQA datasets (HRF-quality, DRIMDB, EyeQ and DeepDRiD) analysed in this study are freely available from their contributors. All experiments were carried out in accordance with relevant guidelines and regulations. Our vessel segmentation model is trained on RETA dataset. One public trained vessel segmentation model is available at https://retabenchmark.org/. Supplementary data of this study can also be found at https://github.com/XingzhengLyu/fractal_dimension_RIQA.
